# Sex composition and FDG-PET prediction of Alzheimer's disease conversion: response to Rudroff

**DOI:** 10.1016/j.jnha.2026.100988

**Published:** 2026-09-26

**Authors:** Ayman S. Alhasan, Mustafa S. Alhasan, James Milburn, Hadeel A. Ghunaim, Mohammad Khalil, Abdullah Almaghraby, Omar Alharthi, Seham Hamoud, Muhammed Amir Essibayi, Yasir Hassan Elhassan, Fabricio Feltrin, Sumit Singh, Dhairya A. Lakhani, Ahmed Y. Azzam

**Affiliations:** a Department of Internal Medicine, College of Medicine, Taibah University, Madinah, Saudi Arabia; b Department of Internal Medicine, College of Medicine, Taibah University, Madinah, Saudi Arabia, Consultant Teleradiologist, Teleradiology Solutions, 22 Llanfair Rd UNIT 6, Ardmore, PA 19003, United States of America; c The University of Queensland Medical School, Ochsner Clinical School, Department of Radiology, Ochsner Clinic Foundation, New Orleans, Louisiana, United States of America; d Faculty of Medicine, King Abdulaziz University, Jeddah, Saudi Arabia; e Department of Pediatrics, Umm Al-Qura University, Makkah, Saudi Arabia; f Department of Pediatrics, Taibah University, Madinah, Saudi Arabia; g Montefiore-Einstein Cerebrovascular Research Lab, Montefiore Medical Center, Albert Einstein College of Medicine, Department of Neurological Surgery, Bronx, NY, United States of America; h Department of Basic Medical Sciences, College of Medicine, Taibah University, Madinah, Saudi Arabia; i Division of Radiology - Neuroradiology, University of Texas Southwestern Medical Center, 5323 Harry Hines Blvd, Dallas, TX 75390, United States of America; j Department of Neuroradiology, Rockefeller Neuroscience Institute, West Virginia University, Morgantown, West Virginia, United States of America; k Department of Neuroradiology, Rockefeller Neuroscience Institute, West Virginia University, Morgantown, West Virginia, United States of America, Division of Global Health and Public Health, School of Nursing, Midwifery and Public Health, University of Suffolk, Ipswich, Suffolk, United Kingdom

Dear Editor,

We thank Rudroff for highlighting the importance of biological sex in FDG-PET research. Our study [1] evaluated baseline FDG-PET as a prognostic biomarker of cognitive decline and disease conversion in ADNI, whereas Rudroff [2] argues that differences in female representation across the cognitively normal, mild cognitive impairment, and Alzheimer's disease groups may have confounded the findings. This concern conflates two distinct populations. Table 1 described the full cohort of 4,732 participants, but the conversion analyses were restricted to 1,136 participants who were all cognitively normal at baseline [1]. Thus, the sex composition of the baseline mild cognitive impairment and Alzheimer's disease groups did not enter the risk set used to estimate the incidence rate ratio of 3.79 or AUC of 0.826. Differences in sex proportions across separate diagnostic strata do not establish confounding or effect modification. Confounding would require sex to be associated with both baseline FDG metabolism and subsequent conversion within the baseline cognitively normal cohort, with material alteration of the FDG estimate after adjustment. Effect modification would require a formal FDG by sex interaction. Our parsimonious models were designed to quantify prognostic information in baseline FDG-PET, not to estimate a causal effect independent of every demographic characteristic.

The concern regarding pooled MetaROI z-score standardization also warrants clarification. A pooled z transformation is a linear rescaling that changes the origin and units while preserving participant ordering. It cannot create an association, rank separation, or discrimination absent from the underlying FDG values. It may affect the portability of fixed thresholds across populations, which is relevant to external validation, but this does not show that standardization generated the observed gradient. The quantitative examples cited by Rudroff [2] were derived from globus pallidus metabolism in COVID-19 survivors and frontal-striatal metabolism in multiple sclerosis and Long COVID. Those diseases, regions, outcomes, and sampling structures are not interchangeable with an Alzheimer-sensitive cortical MetaROI, so their variance estimates cannot be transferred directly to our cohort. Evidence in Alzheimer's disease supports sex-related metabolic heterogeneity, but its direction and magnitude vary by biomarker, region, disease stage, and pathology burden. Tsiknia et al. [3] reported sex modification of associations between plasma p-tau181 and several Alzheimer biomarkers, including glucose metabolism, while Nerattini et al. [4] found greater limbic and frontal hypometabolism in women but greater parietal hypometabolism in men. These findings support interaction testing, not presumption of a uniform sex-driven shift explaining our results.

We agree that sex-adjusted and sex-stratified analyses would strengthen future validation and clinical translation. Because sex was not included in the primary FDG-only models, our study should not be interpreted as proving absence of a sex effect, and the terms "determinant" and "metabolic protection" should be understood as descriptions of prospective prognostic gradients rather than causal effects of modifying metabolism. Future work should evaluate FDG by sex interactions, sex-specific discrimination and calibration, and replication in more diverse independent cohorts. Nevertheless, the correspondence does not demonstrate that sex composition invalidated the associations. Among individuals cognitively normal at baseline, lower Alzheimer-sensitive FDG MetaROI values prospectively identified faster cognitive decline and substantially greater conversion risk, with concordant findings across longitudinal modeling, survival analysis, sensitivity analyses, calibration assessment, and subject-grouped cross-validation [1]. The appropriate conclusion is that sex is an important candidate modifier requiring focused validation, not that it has been shown to account for the observed FDG-PET prognostic signal.

## CRediT authorship contribution statement

Ayman S. Alhasan: Conceptualization, Methodology, Formal Analysis, Investigation, Writing – Original Draft, Writing – Review & Editing, Project Administration; Mustafa S. Alhasan: Conceptualization, Methodology, Formal Analysis, Investigation, Writing – Original Draft, Writing – Review & Editing, Project Administration; James Milburn: Methodology, Validation, Writing – Original Draft, Writing – Review & Editing; Hadeel A. Ghunaim: Investigation, Writing – Review & Editing; Mohammed Khalil: Investigation, Writing – Review & Editing; Abdullah Almaghraby: Investigation, Writing – Review & Editing; Omar Alharthi: Investigation, Writing – Review & Editing; Seham Hamoud: Investigation, Writing – Review & Editing; Muhammed Amir Essibayi: Investigation, Writing – Review & Editing; Yasir Elhassan: Investigation, Writing – Review & Editing; Fabricio Feltrin: Validation, Visualization, Writing – Review & Editing; Sumit Singh: Validation, Visualization, Writing – Review & Editing; Dhairya A. Lakhani: Validation, Visualization, Supervision, Writing – Review & Editing; Ahmed Y. Azzam: Conceptualization, Methodology, Software, Formal Analysis, Data Curation, Writing – Original Draft, Writing – Review & Editing, Visualization, Supervision, Project Administration.

## Declaration of Generative AI and AI-assisted technologies in the writing process

The authors declare that generative AI and AI-assisted technologies were used during the preparation of this manuscript strictly for language editing to refine clarity, grammar, and readability. No AI tools were used in the generation of scientific content, data analysis, interpretation of results, or creation of figures, images, or artwork. The authors reviewed and edited all AI-assisted output and take full responsibility for the content of the published article.

## Declaration of competing interest

The authors declare no conflicts of interest.
